# Invasive predatory fish occupies highest trophic position leading to expansion of isotopic niches in a riverine food web

**DOI:** 10.1002/ecy.70180

**Published:** 2025-09-04

**Authors:** Olivia C. Hodgson, Sydney Stark, Megan K. Schall, Geoffrey D. Smith, Kelly L. Smalling, Tyler Wagner

**Affiliations:** ^1^ Pennsylvania Cooperative Fish and Wildlife Research Unit The Pennsylvania State University University Park Pennsylvania USA; ^2^ Department of Biology Pennsylvania State University Hazleton Hazleton Pennsylvania USA; ^3^ Pennsylvania Fish and Boat Commission, Division of Fisheries Management Bellefonte Pennsylvania USA; ^4^ U.S. Geological Survey New Jersey Water Science Center Lawrenceville New Jersey USA; ^5^ U.S. Geological Survey, Pennsylvania Cooperative Fish and Wildlife Research Unit The Pennsylvania State University University Park Pennsylvania USA

**Keywords:** food web, invasion ecology, *Pylodictis olivaris*, river, trophic dispersion, trophic displacement, trophic disruption hypothesis, trophic position flathead catfish

## Abstract

Invasive species are drivers of ecological change with the potential to reshape the structure and function of terrestrial and aquatic ecosystems. The invasive flathead catfish (*Pylodictis olivaris*) is an opportunistic predator that has established a rapidly growing population in the Susquehanna River, Pennsylvania, USA, since they were first detected in 2002. Although the predatory effects of invasive catfishes on native fish communities have been documented, the effects of invasion on riverine food webs are poorly understood. This study quantified the effects of invasive flathead catfish on the trophic position (TP) and isotopic niche of the river's food web by comparing invaded and non‐invaded sites. In addition to flathead catfish, the food web components examined included crayfish, minnows, and two ecologically and socioeconomically important fish species: the smallmouth bass (*Micropterus dolomieu*) and channel catfish (*Ictalurus punctatus*). We found that flathead catfish occupied the highest TP, with a posterior mean TP of 3.08 (95% credible interval = [2.71, 3.42]), exceeding that of the two resident fish predators, the smallmouth bass and channel catfish. The TP of the resident channel catfish, which occupies a similar ecological niche, declined after flathead catfish invasion. In fact, there was a 0.92 posterior probability that channel catfish TP was lower in invaded sites than at non‐invaded sites. Using a Bayesian bivariate ellipses analysis, we found overwhelming evidence of isotopic niche expansion and overlap for all components of the food web in the presence of flathead catfish. These findings support the “trophic disruption hypothesis,” where an introduced species prompts resident species to change diets in an attempt to avoid competition and predation following invasion. Our results indicate that flathead catfish invasion is altering food web structure and energy flow in a large riverine ecosystem and contributes to the breadth of knowledge regarding how ecosystems may respond to the introduction of a large predatory fish species.

## INTRODUCTION

Invasive species are among the leading drivers of ecological change, reshaping ecosystems and threatening biodiversity across terrestrial and aquatic environments. By altering species interactions, community composition, and ecosystem processes, invasive species often disrupt the balance of native systems, leading to cascading ecological and economic consequences (David et al., 2017; Glassic et al., 2023). Freshwater ecosystems, which are recognized to be among the most imperiled habitats on Earth, are especially vulnerable to invasive species (Cucherousset & Olden, 2011). Invasive predators, in particular, pose a significant threat to invaded ecosystems. Whether in terrestrial or aquatic environments, these species often restructure food webs by preying on native species, outcompeting established predators, and altering energy flows (David et al., 2017; Glassic et al., 2023). This is especially true for predatory fishes, which are strongly associated with the decline and extirpation of species worldwide; through predation and altering the behaviors of resident species at all trophic levels, invasive predatory fishes jeopardize and disrupt the productivity and stability of entire ecosystems (Dick et al., 2013; Gallardo et al., 2016). Predatory fishes have been widely introduced (Franco et al., 2021; Joffe‐Nelson et al., 2023; Kopp et al., 2009) and often perform well outside their native range because they frequently possess characteristics of a successful invader, such as fast growth rates, large body size, habitat generalism, and diet plasticity (David et al., 2017; Glassic et al., 2023).

Many species of catfish (often in the genera *Ictalurus* and *Pylodictis*) possess traits that increase their potential to become invasive outside their native range, including their role as apex predators, opportunistic feeding, and tolerance to a wide range of environmental conditions (Belkoski et al., 2021). This is true for flathead catfish (*Pylodictis olivaris*)—a large piscivorous apex predator in the family Ictaluridae that thrives in a variety of different environments, with a nonselective feeding behavior and the ability to grow to large sizes (Schmitt et al., 2019). Although flathead catfish are native to the Mississippi River, Mobile River, and Rio Grande drainage areas (Fuller et al., 1999; Irwin, 1999), they have been introduced into many Atlantic coast rivers and areas of the western United States (Fuller et al., 2019). The flathead catfish is now recognized as an invasive predatory fish throughout its introduced range in the United States (Smith et al., 2021).

Studies have documented the negative impacts introduced flathead catfish can have on resident species, including sunfishes *Lepomis* spp., black bass *Micropterus* spp., other catfishes, as well as species of conservation concern, such as Atlantic sturgeon (*Acipenser oxyrinchus*), American eels (*Anguilla rostrata*), and various diadromous *Alosa* spp. (Baumann & Kwak, 2011; Bonvechio et al., 2009; Pine et al., 2007; Schmitt et al., 2019). In addition to fishes, flathead catfish will consume aquatic insects and crayfish, although negative impacts have not been quantified in invertebrates (Belkoski et al., 2021; Stark et al., 2024). In introduced habitats, flathead catfish are not restricted to consuming prey items they recognize and will consume organisms that do not exist within the flathead catfish's native range (Pine et al., 2005; Sakaris et al., 2006). Thus, the stomach contents of flathead catfish reflect prey availability rather than preference (Belkoski et al., 2021; Schmitt et al., 2019; Stark et al., 2024). Flathead catfish have the potential to decimate native and recreational fisheries in river systems where they have become established after their introduction or invasion from nearby systems, and they have the potential to disrupt the structure and function of riverine food webs. Although studies have investigated the diets of flathead catfish, little is known about their effects on the food webs they invade.

Stable isotope analysis (SIA) is a widely used tool that can explain patterns within a food web, highlighting links between trophic positions (TPs), as well as the breadth and overlap of trophic niches (Layman et al., 2007; Vander Zanden et al., 1999). SIA is also useful for studying invasion ecology, such as investigating trophic reorganization and trophic overlap between introduced and resident species (Jackson et al., 2012). Specifically, δ^15^N (15N/14N ratio) is useful for determining an organism's TP and potential shifts in TP following invasion, while δ^13^C (13C/12C ratio) is useful for illustrating energy inputs in the system and energy flow between primary producers and consumers (Post, 2002; Quintana et al., 2023). By quantifying the dispersion of δ^13^C and δ^15^N values in bivariate space, SIA can describe isotopic niche space, providing insights into consumed trophic resources. This approach allows for the calculation of community niche metrics (Layman et al., 2007) and the evaluation of isotopic niche characteristics related to resource use, trophic diversity, and niche size, making it particularly valuable for evaluating diet overlap, feeding competition, and the displacement of native species by invaders (Harris et al., 2022; Haubrock et al., 2020). Resource use can be inferred from the isotopic niche space occupied by an organism, and an overlap of the niche space between organisms indicates potential competition for resources (Lamb et al., 2021; Pagani‐Núñez et al., 2018; Quintana et al., 2023).

The goal of this research was to quantify the impacts of an introduced flathead catfish population on select components of the Susquehanna River, Pennsylvania (PA), food web using SIA. The Susquehanna River is the largest river within the United States that drains into the Atlantic Ocean and the largest tributary to the Chesapeake Bay, stretching through three states and spanning approximately 715 km and draining 71,224 km^2^. The West Branch Susquehanna River (hereafter referred to as the West Branch)—the largest tributary to the Susquehanna River—is approximately 391 km long and drains 17,730 km^2^ in central PA (Smith et al., 2021; Zhang et al., 2010). First detected in 2002, the flathead catfish quickly established a viable population that grew rapidly and has continued a northward expansion through the Susquehanna River system and its tributaries (Brown et al., 2005; Massie et al., 2018; Smith et al., 2021). The establishment of this predatory species in the Susquehanna River has raised concerns over the potential ecological impacts to this diverse riverine food web (Belkoski et al., 2021; Schmitt et al., 2019). Flathead catfish consume a wide variety of prey in this system, including species of recreational and conservation importance that may suffer population declines from prolonged exposure to predation (Stark et al., 2024).

Although flathead catfish have expanded northward in the mainstem of the Susquehanna River, at the time of this study (2022–2023), they had not yet invaded the West Branch in the north‐central region of the state (Smith et al., 2021). Aside from flathead catfish, the West Branch is an ecologically analogous tributary to the mainstem of the Susquehanna River. Many of the species in both river sections are common warmwater fishes and invertebrates that fill similar ecological niches (Dunlap et al., 2011; Smith et al., 2021). This presents a natural experiment, allowing us to compare food webs between invaded and non‐invaded sites within the same river system. By examining these differences, we can assess how flathead catfish invasion influences food web dynamics and enhance our understanding of how an invasive apex predator influences riverine food webs, ecosystem services, and local economies. The specific objectives were to (1) elucidate the TP of flathead catfish and other organisms within the food web and determine whether the TP of resident fishes shifted in the presence of flathead catfish and (2) quantify possible changes (expansions or contractions) in isotopic niche space resulting from invasion. The findings of this study provide novel insights into the effects of a predatory invader on a large riverine food web.

## METHODS

### Study area

**FIGURE 1:**
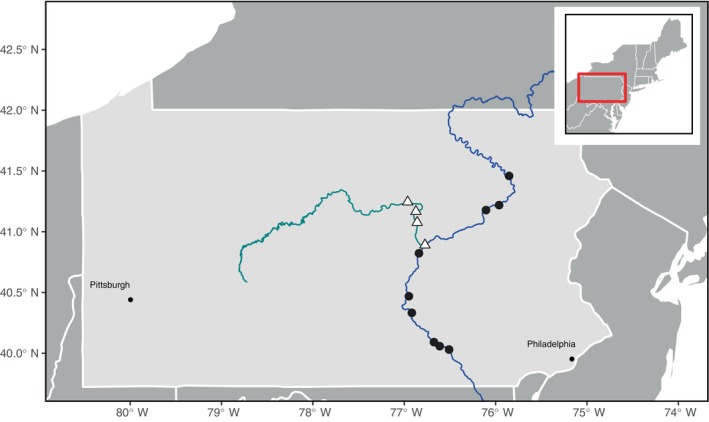
Location of study sites on the Susquehanna and West Branch Susquehanna rivers, Pennsylvania. The blue line (running north to south) represents the main stem of the Susquehanna River, while the teal line (running west to east) represents the West Branch. The white triangles indicate sampling locations invaded by flathead catfish (*Pylodictis olivaris*), and the dark gray triangles indicate locations where flathead catfish have not yet been detected.

Our study area consisted of the Susquehanna River mainstem, which flows north to south in eastern central PA, and the West Branch, a tributary of the Susquehanna River that flows west to east in central PA (Figure 1). Fisheries surveys performed by the state fisheries management agency, the Pennsylvania Fish and Boat Commission, confirmed that flathead catfish were present in the Susquehanna River and absent from the West Branch (Smith et al., 2021). River sites were sampled along the Susquehanna and West Branch rivers for components of the food web (see *Data collection*) in 2022 and 2023 and were chosen to include both sites that were invaded (*n* = 9) and not invaded (*n* = 4) by flathead catfish. This sampling scheme set up a natural experiment to compare food webs between invaded and non‐invaded river sites.

### Riverine food web

Several components of the riverine food web were sampled to assess the potential effects of flathead catfish invasion on food web structure and energy flow. The components of the food web included primary producers (e.g., benthic algae), crayfish, minnows, and three ecologically and socioeconomically important predatory fishes: the focal invasive species, the flathead catfish, and two naturalized species—the channel catfish (*Ictalurus punctatus*) and smallmouth bass (*Micropterus dolomieu*). Smallmouth bass were introduced to the Susquehanna River in the late nineteenth century, and channel catfish were introduced sometime between the late 1890s and late 1910s, both of which had over a century to establish self‐sustaining populations, adapt to local conditions, and become fully integrated (naturalized) into the Susquehanna River ecosystem (Schall et al., 2020). Channel catfish, smallmouth bass, minnows, and crayfish were selected as focal species because a previous diet analysis within the Susquehanna River showed that these species are important prey for flathead catfish (Stark et al., 2024), and this predation could have implications for the structure and energy flow within the Susquehanna River food web. In addition, channel catfish occupy a similar ecological niche as flathead catfish and therefore may be negatively impacted by competition (and not just predation). Smallmouth bass are a predatory fish and an important recreational fishery in the Susquehanna River Basin (Schall et al., 2023). There may be substantial ecological and socioeconomic implications if smallmouth bass are negatively affected by flathead catfish invasion. Crayfish and minnows were not only collected to represent lower trophic levels but are also the two most common prey for flathead catfish within the study system (Stark et al., 2024). Because our focal components of the food web have been shown to be important prey of flathead catfish and there is the potential for interspecific competition among some components, we predicted that our sampled food web would show a particularly strong isotopic signature in response to flathead catfish invasion.

### Data collection

Sampling occurred in the summers of 2022 and 2023 (between July and September) when water temperatures were >18°C to maximize sampling efficiency for flathead catfish. A variety of sampling approaches and gear types were used to sample different trophic levels. Benthic algae and crayfish were collected by hand. Minnows and smallmouth bass were collected using a boat‐mounted ETS Electrofishing System, LLC MBS‐1 electrofisher (Madison, Wisconsin) with high‐frequency (60 Hz) pulsed direct current at a 25%–30% duty cycle, targeting 16–20 peak amperes. Suitable shoreline habitat was targeted for both species within the same pool where flathead catfish were collected. The sample sites included areas where flathead catfish were present (invaded) or absent (not invaded) based on historical data and surveys by the PA Fish and Boat Commission (Smith et al., 2021). The minnows were collected opportunistically, and it was not possible to sample minnows of the same species at every site; however, all minnows (Appendix S1: Table S1) were ecologically similar in terms of their feeding behavior and therefore trophic niche, consuming a variety of detritus, algae, and small aquatic invertebrates (Burress et al., 2016). Channel catfish and flathead catfish were collected using baited hoop nets, which included four sequential hoop nets (three nets per series, each 4.9 m long with 38.1‐mm mesh and seven 1.2‐m hoops that taper toward the cod end [Miller Net Company, Inc., Memphis, TN]). Nets were set at each sample site for 72 h, baited with 1 kg of cheese logs (Boatcycle Co., Inc., Henderson, TX) per net. Once caught, all fish were euthanized using a lethal dose of tricaine methanesulfonate (TMS, MS‐222). Individual length (in millimeters) was recorded as the total length for fish and the carapace length for crayfish. All fish sampling and handling procedures were approved by the Pennsylvania State University Animal Care and Use Committee (PROTO202102088; PROTO201901210).

### Stable isotope analysis

Benthic algae, crayfish, minnows, channel catfish, smallmouth bass, and flathead catfish samples were analyzed for δ^15^N and δ^13^C determination. For crayfish and fish species, stable isotopes were determined for muscle tissue. All samples were oven‐dried at 60°C for approximately 48 h and ground to a fine powder using a mortar and pestle. Stable isotope samples were sent to The Pennsylvania State University Core Facilities and The Michigan State University Stable Isotope laboratories for isotope determination. Because the δ^15^N values cannot be directly compared between consumers from different sampling locations due to potential differences in baseline δ^15^N signatures (Post, 2002; Vander Zanden et al., 1997, 1999), primary producer (algae) samples were used to correct the δ^15^N values of organisms. Furthermore, because δ^13^C fractionates between different tissues, the C:N ratios for all consumers were calculated and found to be less than 3.5, indicating that lipid correction for δ^13^C values was not necessary (Skinner et al., 2016).

### Statistical analysis

#### Modeling stable isotope variation

To evaluate how stable isotopes varied between consumers, as a function of organism size (i.e., length), and between invaded and non‐invaded river sites, we fitted a Bayesian hierarchical regression model as follows:

(1) $${\mathrm{SI}}_{\mathrm{ijk}}˜N\left(\mu_{\mathrm{ijk}},\sigma^{2}\right)$$

(2) $$\mu_{\mathrm{ijk}}=\alpha_{j}+\beta_{1j}\times {\text{length}}_{\mathrm{ijk}}+\beta_{2j}\times {\text{invasion}}_{k}+\gamma_{k}$$

where ${\mathrm{SI}}_{\mathrm{ijk}}$ is one of the stable isotope values, δ^15^N (corrected for baseline; $\delta^{15}N_{\text{adjusted}}$) or δ^13^C, from individual *i* of species *j* from site *k*. ${\mathrm{SI}}_{\mathrm{ijk}}$ was assumed to be normally distributed with an expected value $\mu_{\mathrm{ijk}}$ that was modeled as a function of species‐specific intercepts ($\alpha_{j}$), length, and whether or not fish came from an invaded or non‐invaded site, where $\beta_{1j}$ and $\beta_{2j}$ are species‐specific length and invasion status effects, respectively. A site random effect was also included, $\gamma_{k}$, to account for multiple individuals of each species being sampled from each site, and was assumed normally distributed with a mean 0 and variance $\tau^{2}$ (i.e., $\gamma_{k}˜N\left[0,\tau^{2}\right]$). Models were fit using the R package rstanarm (Goodrich et al., 2024) using default priors on all parameters. By default, the R package rstanarm uses weakly informative priors designed to help stabilize computation by internally adjusting the scales of the priors. A Gaussian prior was used for intercept and slope parameters and an exponential prior was used for the residual and random effects SD. To obtain information on the exact priors used—after internal adjustments were made—refer to the data and code release that accompany this paper (https://doi.org/10.5066/P16KW2QC).

Three Markov Chain Monte Carlo (MCMC) simulations were run for 1500 iterations, where 750 iterations of each MCMC chain were discarded as burn‐in, leaving 2250 total samples for posterior inference. Models were visually assessed for convergence using trace plots and examination of the potential scale reduction factor, $\hat{R}$ (Brooks & Gelman, 1998). Predictor variable significance was determined by examining whether the 95% credible interval (CI) of the parameter included zero. Stable isotope estimates are reported as posterior means and 95% CIs. We also report the probability of specific effects differing (being greater or smaller) from each other (described by the posterior distribution; the posterior probability [PPR]) to take advantage of the distribution of possible values for estimated effects, rather than relying purely on point estimates and CIs. These posterior probabilities provide additional evidence of the potential ecological significance of statistical inferences (Makowski et al., 2019). We considered a PPR of >0.85 as being ecologically meaningful; however, we avoid imposing a specific threshold to determine whether a PPR is biologically significant because we purposely avoid arbitrary “significance levels” with this statistic. Rather, decisions of what PPR magnitude is ecologically significant are context‐dependent and will reflect the amount of uncertainty one is willing to accept for a given inference.

Equations (1) and (2) quantify potential δ^15^N enrichment that can occur as individuals grow (i.e., the length effect) and for differences between invaded and non‐invaded sites. Taking into account this length effect is important because δ^15^N often becomes enriched in fishes over time (Post et al., 2000; Vander Zanden et al., 1999). In our study system, there was the potential for fishes that were sampled from invaded and non‐invaded sites to differ in average length; such a “length effect” could obscure potential differences in δ^15^N resulting from flathead catfish invasion (i.e., if length was not controlled for, then we could be quantifying a “length effect” instead of an “invasion” effect). Therefore, when comparing stable isotope values among invaded and non‐invaded sites, we controlled for fish length by predicting values for an average‐sized individual.

#### Estimating TP

In addition to understanding variation in δ^15^N and δ^13^C values among invaded and non‐invaded sites, we were also interested in estimating the TP for each component of the food web and whether or not there was evidence that TP had shifted as a result of flathead catfish invasion. Similar to how we controlled for potential size effects when modeling stable isotope variation, we also controlled for any potential length effect that could influence TP inferences. Specifically, TP was derived using the posterior samples from the δ^15^N model (Equations 1 and 2) for an average‐sized organism at both invaded and non‐invaded sites. TP was calculated using the equation adapted from Vander Zanden et al. (1997), where $\mathrm{TP}=\left[\left(\delta^{15}N_{\text{consumer}}-\delta^{15}N_{\text{baseline}}\right)/3.4\right]+1$, where 3.4 was the assumed trophic enrichment of δ^15^N and the “+1” is the TP of the baseline (algae) used to correct δ^15^N values. In our case, $\left(\delta^{15}N_{\text{consumer}}-\delta^{15}N_{\text{baseline}}\right)$, which is the δ^15^N adjusted for baseline ($\delta^{15}N_{\text{adjusted}}$), was the posterior predicted values of $\delta^{15}N_{\text{adjusted}}$ from Equation (1) for an average‐sized organism (fish species or crayfish). This approach provided a posterior distribution of TP for each species that controlled for potential effects of length at both invaded and non‐invaded sites and propagated uncertainty through to the derived TP values.

#### Isotopic niche space

We estimated the isotopic niche area for each component of the food web at both invaded and non‐invaded sites using Bayesian standard ellipse area (SEAb) and standardized ellipse area corrected (SEAc) for sample size (Jackson et al., 2011) using the SIBER R package (Jackson & Parnell, 2023). Stable isotope ellipses are a widely used tool in ecological studies to represent the isotopic niche of a species or community in δ^13^C and δ^15^N bivariate space. These ellipses are used to capture the isotopic niche by encompassing approximately 40% of the data, providing a robust estimate of trophic diversity and resource use while accounting for variation and sample size (Jackson et al., 2011). SEAb and SEAc estimate the area of this niche, with SEAc correcting for differences in sample size to allow more accurate comparisons between groups.

To assess whether isotopic niches expanded or contracted in the presence of flathead catfish, we calculated six Layman metrics (Layman et al., 2007), which describe different aspects of trophic structure and niche space. These include (1) total area, representing the overall niche width; (2) δ^13^C range, indicating the diversity of basal energy sources; (3) δ^15^N, reflecting the vertical structure of the food web and TP diversity; (4) mean distance to centroid, a measure of the average trophic diversity within the niche; (5) mean nearest neighbor distance, quantifying the density and evenness of species within the niche space; and (6) SD of nearest neighbor distance, representing the evenness of trophic diversity. These metrics were selected because they collectively provide a comprehensive view of niche breadth, trophic diversity, and resource use within the invaded and non‐invaded food webs. We then measured the proportional change in these metrics between invaded and non‐invaded sites. All analyses were performed using the programming environment R (R Core Team, 2024).

## RESULTS

### Catch summary

A total of 279 fishes and 64 crayfish were collected for SIA, including 79 flathead catfish from the Susquehanna River. The mean (±SD) length of flathead catfish was 725 mm (±180; Table 1). For other species, the overall size varied across invasion levels. Smallmouth bass from invaded sites (*n* = 28) were generally larger than those from non‐invaded sites (*n* = 17), with a PPR of 15% that those collected from non‐invaded habitats were larger. In contrast, channel catfish at invaded sites (*n* = 81) were smaller, with a PPR of 97% that fish collected from non‐invaded sites where larger. Channel catfish from invaded sites averaged 564 ± 88 mm, while those from non‐invaded sites (*n* = 32) averaged 608 ± 76 mm (Table 1). Minnows, comprising nine species (Appendix S1: Table S1), were collected from invaded sites (*n* = 25) and non‐invaded sites (*n* = 17), with a PPR of only 6% that the minnows from non‐invaded sites were larger than those from invaded sites. Mean lengths were 124 ± 78 mm at invaded sites and 78 ± 37 mm at non‐invaded sites (Table 1). No clear size difference was observed for crayfish between invaded (*n* = 44) and non‐invaded sites (*n* = 20; PPR = 50%), with lengths being similar among all sites (Table 1). Crayfish species included the native Allegheny crayfish (*Faxonius obscurus*) and introduced rusty crayfish (*Faxonius rusticus*).

**TABLE 1:** Number (*n*) of samples analyzed for stable isotopes (δ^15^N and δ^13^C) and total lengths (minimum [Min], maximum [Max], median, mean, and SD, in millimeters) of flathead catfish (*Pylodictis olivaris*), channel catfish (*Ictalurus punctatus*), smallmouth bass (*Micropterus dolomieu*), minnows, and crayfish in Susquehanna River, USA.

Species	Invaded sites					
	*n*	Total lengths				
		Min	Max	Median	Mean	SD
Flathead catfish	79	385	1119	723	725	180
Channel catfish	81	371	728	575	564	88
Smallmouth bass	28	157	479	284	307	85
Minnow	25	64	305	88	124	78
Crayfish	44	12	44	30	29	7

Species	Non‐invaded sites						PPR
	*n*	Total lengths					
		Min	Max	Median	Mean	SD	
Flathead catfish							
Channel catfish	32	454	740	610	608	76	0.97
Smallmouth bass	17	153	392	282	272	80	0.15
Minnow	17	44	177	62	78	37	0.06
Crayfish	20	22	42	29	29	4	0.50

*Note*: Data are presented for locations both invaded and not invaded by flathead catfish, along with the posterior probabilities (PPR) that individuals from non‐invaded habitats were larger than those from invaded habitats.

### δ^13^ C

**FIGURE 2:**
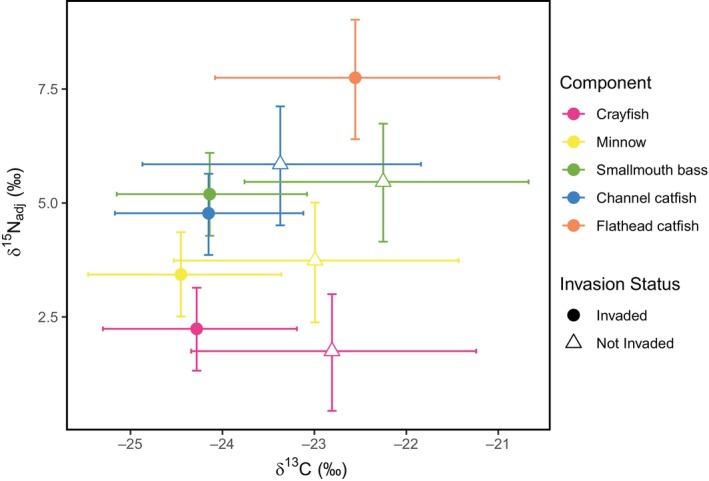
Estimated δ^15^N and δ^13^C values for each of the five sampled components of the Susquehanna River, Pennsylvania, food web based on flathead catfish (*Pylodictis olivaris*) invasion status (Invaded or Not Invaded). Points represent posterior means and bars are 95% credible intervals. The estimated stable isotope values are for organisms of average length (flathead catfish = 725 mm, channel catfish (*Ictalurus punctatus*) = 576 mm, smallmouth bass (*Micropterus dolomieu*) = 393 mm, minnows = 105 mm, and crayfish = 29 mm).

Posterior mean δ^13^C values (per mille; ‰) varied little among sites. At sites without flathead catfish, δ^13^C values showed minimal variation among species, ranging from −23.36 (95% CI [−24.94, −21.89]; Figure 2) for channel catfish to −22.24 (95% CI [−23.83, −20.65]; Figure 2) for smallmouth bass. In contrast, at sites with flathead catfish, δ^13^C was highest in flathead catfish, with a mean value of −22.54 (95% CI [−24.18, −21.03]). Among the remaining species, δ^13^C values were relatively similar, ranging from −24.53 (95% CI [−25.64, −23.44]; Figure 2) to −24.22 (95% CI [−25.32, −23.14]; Figure 2) for minnows and smallmouth bass. Because there is little trophic enrichment of δ^13^C (Post, 2002), δ^13^C can indicate dietary sources of carbon in lotic food webs. The relatively similar δ^13^C among components of the food web suggests that they derive carbon from similar basal resources; however, there is evidence of expanding basal resources in the presence of flathead catfish (see *Isotopic space and position*; Pingram et al., 2012).

### δ^15^ N and TP

The posterior mean δ^15^N values (per mille;) followed expectations based on the ecology of the food web components and also increased with increasing length for flathead catfish, channel catfish, and smallmouth bass (Appendix S1: Figure S1). Posterior mean δ^15^N values corrected for baseline differed between invaded and non‐invaded sites. At sites where flathead catfish were absent, the posterior mean δ^15^N value was highest for channel catfish (5.87, 95% CI [4.62, 7.18]), followed by smallmouth bass (5.48, 95% CI [4.22, 6.76]), minnows (3.76, 95% CI [2.47, 5.08]), and crayfish (1.77, 95% CI [0.49, 3.05]; Figure 2). At sites where flathead catfish were present, flathead catfish had the highest posterior mean δ^15^N value (7.77, 95% CI [6.55, 9.07]), followed by smallmouth bass (5.20, 95% CI [4.22, 6.04]), channel catfish (4.78, 95% CI [3.94, 5.64]), minnows (3.44, 95% CI [2.55, 4.29]), and crayfish (2.25, 95% CI [1.41, 3.07]; Figure 2).

**FIGURE 3:**
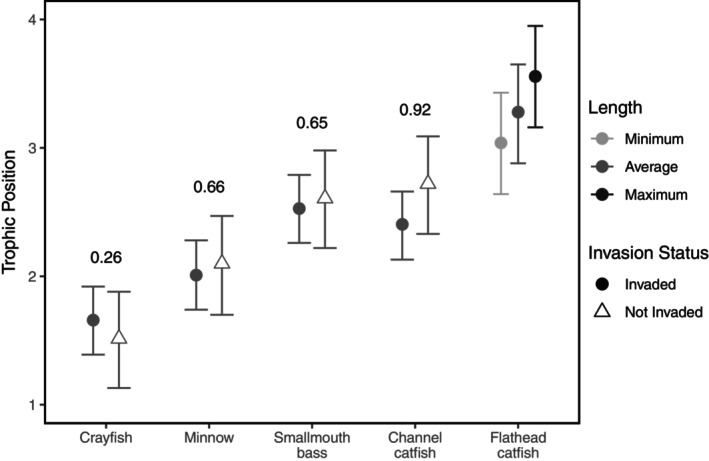
Estimated trophic positions for each of the five sampled components of the Susquehanna River, Pennsylvania, food web based on flathead catfish (*Pylodictis olivaris*) invasion status (Invaded or Not Invaded). Points represent posterior means, and bars are 95% credible intervals. Posterior probabilities that the trophic position of each component is higher at non‐invaded sites than invaded sites are reported above the respective posterior means. Estimated trophic positions for flathead catfish are provided for the minimum (385 mm), average (725 mm), and maximum (1119 mm) sized flathead catfish captured in this study. For other food web components, estimated trophic positions correspond to average‐sized individuals (channel catfish (*Ictalurus punctatus*) = 576 mm, smallmouth bass (*Micropterus dolomieu*) = 393 mm, minnows = 105 mm, and crayfish = 29 mm).

As expected, length‐adjusted TP estimates followed the same pattern as the δ^15^N values from which they were derived. In sites without flathead catfish, channel catfish occupied the highest TP (posterior mean TP = 2.72, 95% CI [2.36, 3.11]), followed by smallmouth bass (TP = 2.61, 95% CI [2.24, 2.99]), minnows (TP = 2.11, 95% CI [1.73, 2.49]), and crayfish (TP = 1.52, 95% CI [1.15, 1.90]; Figure 3). For invaded sites, flathead catfish of all lengths occupied the highest TP. The largest flathead catfish sampled in this study (1119 mm) occupied a TP of 3.32 (95% CI [2.94, 3.68]). There was a 100% PPR that large flathead catfish occupied a greater TP compared to channel catfish and smallmouth bass at invaded sites. Average‐sized flathead catfish (725 mm) had a TP of 3.08 (95% CI [2.71, 3.42]), whereas the smallest sized (385 mm) flathead catfish had a TP of 2.86 (95% CI [2.48, 3.21]; Table 1; Figure 3).

In the presence of flathead catfish, the length‐adjusted TP estimate of smallmouth bass occupied the next highest TP (TP = 2.53, 95% CI [2.27, 2.78]; Table 1; Figure 3), while the smallmouth bass TP in non‐invaded habitats was higher (TP = 2.61, 95% CI [2.24, 2.99]; PPR of not invaded TP > invaded TP = 0.65). In contrast, after controlling for length differences, channel catfish occupied a lower TP (TP = 2.41, 95% CI [2.16, 2.66]; Figure 3) when flathead catfish were present compared with non‐invaded habitats (posterior mean TP = 2.72, 95% CI [2.36, 3.11]; PPR of not invaded TP > invaded TP = 0.92). Minnows also had a lower TP in invaded habitats (TP = 2.01, 95% CI [1.75, 2.26]; Figure 3) compared with non‐invaded habitats (TP = 2.11, 95% CI [1.73, 2.49]; PPR of not invaded TP > invaded TP = 0.66). Conversely, crayfish occupied a higher TP in invaded habitats (TP = 1.66, 95% CI [1.41, 1.90]; Figure 3) than their counterparts in non‐invaded habitats (TP = 1.52, 95% CI [1.15, 1.90]; PPR of not invaded TP > invaded TP = 0.26).

### Isotopic space and position

**FIGURE 4:**
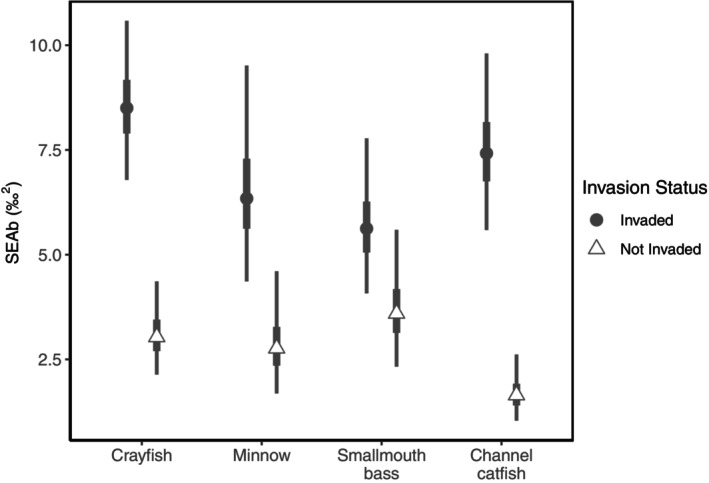
Bayesian standard ellipse areas (SEAb) estimates for components of the Susquehanna River, Pennsylvania, food web at sites invaded and not invaded by flathead catfish (*Pylodictis olivaris*). Points are posterior modes; thick and thin lines are 50% and 95% credible intervals, respectively.

**FIGURE 5:**
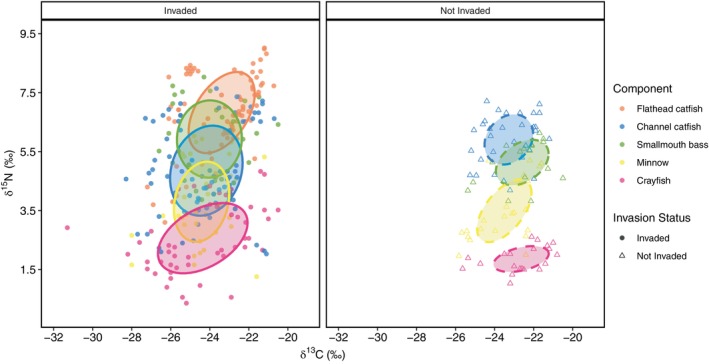
Standard ellipse areas corrected (SEAc) for sample size for components of the Susquehanna River, Pennsylvania, food web sites invaded (left panel; $n_{\text{site}}=9$; $n_{\text{sample}}=348$) and not invaded (right panel; $n_{\text{site}}=4$; $n_{\text{sample}}=128$) by flathead catfish (*Pylodictis olivaris*). Food web components include channel catfish (*Ictalurus punctatus*), smallmouth bass (*Micropterus dolomieu*), minnows, and crayfish.

Results of a Bayesian bivariate ellipses analysis using δ^15^N and δ^13^C indicated, overwhelmingly, niche expansion and increased isotopic overlap for all components of the food web in the presence of flathead catfish. For fish species, SEAb increased by 135% (for minnows) to 270% (for channel catfish) in invaded riverine habitats (Table 2; Figure 4). This drastic expansion in ellipses area led to greater overlap of the isotopic niche among all components of the food web (Table 2; Figure 5). Based on the average percent increase in Layman metrics from non‐invaded to invaded sites, crayfish (average percent change = 202%) and channel catfish (average percent change = 81%) showed the largest niche expansion when comparing invaded and non‐invaded sites. All other Layman metrics increased from non‐invaded to invaded habitats, except for mean nearest neighbor distance for channel catfish and SD of nearest neighbor distance for smallmouth bass, providing further support for niche expansion occurring in habitats invaded by flathead catfish (Table 2).

**TABLE 2:** Layman metrics and percent change in Layman metrics between river sites invaded and not invaded by flathead catfish (*Pylodictis olivaris*) in the Susquehanna River.

Metric	Non‐invaded sites	Invaded sites	% change
Channel catfish (*Ictalurus punctatus*)			
Mean distance to centroid	1.38	2.11	53.25
Mean nearest neighbor distance	0.39	0.32	−19.09
SD of nearest neighbor distance	0.20	0.23	11.43
Total area	9.55	35.30	269.51
δ^13^C range	4.06	7.59	86.95
δ^15^N range	3.02	5.61	85.76
Smallmouth bass (*Micropterus dolomieu*)			
Mean distance to centroid	1.29	1.96	52.61
Mean nearest neighbor distance	0.48	0.53	11.42
SD of nearest neighbor distance	0.37	0.23	−39.53
Total area	6.71	16.27	142.30
δ^13^C range	4.87	6.10	25.26
δ^15^N range	2.31	4.16	80.09
Minnow			
Mean distance to centroid	1.51	1.68	11.08
Mean nearest neighbor distance	0.39	0.45	15.47
SD of nearest neighbor distance	0.36	0.40	11.83
Total area	10.22	24.02	135.10
δ^13^C range	4.23	6.80	60.76
δ^15^N range	3.24	4.77	47.22
Crayfish			
Mean distance to centroid	1.20	2.30	92.63
Mean nearest neighbor distance	0.31	0.46	50.25
SD of nearest neighbor distance	0.14	0.47	237.51
Total area	4.42	27.74	527.52
δ^13^C range	4.84	10.80	123.14
δ^15^N range	1.58	4.47	182.91

*Note*: The values are shown for each metric in units of per mille (‰). The proportional change between invaded and non‐invaded sites is shown as percent change, with positive values indicating an increase in invaded sites compared with non‐invaded sites.

## DISCUSSION

We found that flathead catfish occupy the highest TP and are an apex predator in habitats of the Susquehanna River it has invaded, similar to other studies documenting its apex predator role in both native and introduced ranges (Pine et al., 2005; Sakaris et al., 2006; Schmitt et al., 2019). We also found strong evidence that the introduction of flathead catfish into a large riverine ecosystem resulted in changes in some aspects of the resident food web. The observed changes were consistent with the “trophic disruption hypothesis” (Wainright et al., 2021), whereby an introduced species prompts resident species to change their diets in an attempt to avoid competition and predation after invasion. Wainright et al. (2021) describes two primary mechanisms that contribute to trophic disruption: trophic displacement, defined as switching prey types, and trophic dispersion, characterized by an increase in diet variability.

In the Susquehanna River, flathead catfish appear to cause trophic displacement, a pattern consistent with findings from other predator invasion studies. Wainright et al. (2021) found evidence of trophic displacement, whereby in the presence of lake trout (*Salvelinus namaycush*), native bull trout (*Salvelinus confluentus*, a species filling a similar ecological niche as the lake trout) shifted from an apex predator in non‐invaded lakes to a mesopredator role in invaded lakes. We also observed TP displacement of an ecologically similar species post‐invasion, with evidence that channel catfish were displaced (to a lower TP) from their reference, non‐invaded TP. This displacement could be the result of changes in behavior to avoid predation, a pattern supported by a concurrent diet study that found channel catfish were frequently prey for flathead catfish (Stark et al., 2024). Similarly, in Eastern Canada, non‐native rainbow trout (*Oncorhynchus mykiss*) exploited prey and other resources used by the native Atlantic salmon (salar; Blanchet et al., 2007, 2008), and in New Zealand, the non‐native brown trout (*Salmo trutta*) displaced a native top predator, river galaxias (*Galaxias vulgaris*), with effects cascading to lower trophic levels (McIntosh & Townsend, 1995, 1996; Townsend & Crowl, 1991). Trophic displacement has also been observed in terrestrial systems. A study in southern California's Channel Islands found the introduction of the golden eagle (*Aquila chrysaetos*) forced the island fox (*Urocyon littoralis*), a naive prey item, into nocturnal hunting activity that increased competition with the island spotted skunk (*Spilogale gracilis amphiala*) at a lower TP (Roemer et al., 2002).

Although trophic displacement has been documented in several systems after the establishment of an invasive species (Baer et al., 2022; Tillberg et al., 2007), trophic dispersion is often more challenging to characterize (Gozlan et al., 2010). Using SEAs, our study and Wainright et al. (2021) both found strong evidence of trophic dispersion across food web components in response to predator invasion. In the case of Wainright et al. (2021), dispersion was greatest midinvasion, which they report as ~25 years post‐invasion, a similar timeline for flathead catfish in the Susquehanna River system, which were first observed in 2002 (Smith et al., 2021). This observation of first occurrence has since been supported by demographic history modeling using microsatellite markers (Waraniak et al., 2024). Our results revealed that in invaded habitats, crayfish, minnows, smallmouth bass, and channel catfish in the Susquehanna River food web expanded their isotopic niches and niche overlap significantly increased, suggesting an increase in resource sharing and the potential for more competition throughout the food web (Bearhop et al., 2004; Harris et al., 2022; Quintana et al., 2023). These patterns, which are supported by the expansion in the standard ellipse areas of δ^13^C observed across our focal species, suggest a change in resource use (Vander Zanden et al., 1999), indicating that the species sampled in the Susquehanna River are drawing upon different resources in the habitats they share with flathead catfish to continue maintaining energy requirements.

Niche expansion of resident species is a recurring pattern in response to the invasion of an apex predator. Among salmonids, a study performed by Glassic et al. (2023) found that the isotopic niche of Yellowstone cutthroat trout (*Oncorhynchus clarkii bouvieri*) expanded with an increased abundance of the non‐native apex predator, lake trout, in Yellowstone National Park, Wyoming, USA, as did the niche overlap between the two fishes. In Lake Victoria, the introduction of the non‐native top predator, Nile perch (*Lates niloticus*), led to an increase in niche breadth and dietary overlap among the “resilient native” cyprinid (*Rastrineobola argentea*), *R. argentea*, and native cichlids (Campbell et al., 2003; Ojwang et al., 2004; Sharpe & Chapman, 2014). Similar patterns have been documented in terrestrial systems. In the Caribbean, the introduction of the predatory curly‐tailed lizard (*Leiocephalus carinatus*) increased the diet overlap and competition between the brown (*Anolis sagrei*) and green anoles (*Anolis smaragdinus*) (Pringle et al., 2019). A SIA study in Karoo National Park, South Africa, found a dietary niche expansion in the dominant predator, black‐backed jackals (*Canis mesomelas*), following the reintroduction of the apex predator, the lion (*Panthera leo*), to the reserve (Codron et al., 2018).

These examples call attention to the broader pattern of how resident species respond to the arrival of a new apex predator by expanding their dietary niches and increasing resource overlap. This response is observed in the Susquehanna River following flathead catfish invasion. The magnitude of trophic dispersion and displacement was greatest for crayfish and channel catfish. Catfish in the family Ictaluridae are often the most affected during the invasion by other catfishes (Belkoski et al., 2021; Guier et al., 1984; Pine et al., 2007) because the ecological impacts incurred by non‐native predators are strongest when the invader and resident species are related or fill similar functional roles (Dick et al., 2017; Li et al., 2015; Ricciardi & Atkinson, 2004). When an introduced species is better at exploiting resources used by a resident species, the resident species will often need to expand its trophic niche to continue to meet energy demands (Bolnick et al., 2010; Svanbäck & Bolnick, 2007; Tran et al., 2015).

## CONCLUSIONS

In the Susquehanna River, PA, flathead catfish are apex predators that have altered food web structure and isotopic niche space, with effects varying among trophic levels from predatory fishes to macroinvertebrates. Our findings suggest that the resident species sampled in the Susquehanna River are changing resource use in the habitats they share with flathead catfish, leading to niche expansion and greater interspecific competition. Continued monitoring will help clarify potential population‐level changes of resident species that may occur as a result of flathead catfish invasion. Previous research has demonstrated the transformative effects of flathead catfish on resident communities (Bonvechio et al., 2009; Guier et al., 1984; Pine et al., 2007). Fuller et al. (1999) argued that flathead catfish introductions were the most harmful of all fish introductions in the United States. Our results contribute to a broader understanding of how flathead catfish invasions influence food webs and offer insight for predicting how flathead catfish may impact other river ecosystems invaded by this apex predator. As aquatic invasive species continue to spread throughout the world, understanding their ecological consequences is important to help guide conservation and management efforts. This is especially true for invasive fishes, which pose unique challenges due to their role in altering predator–prey dynamics. This study enhances our understanding of how non‐native predators influence the food webs of the ecosystems they invade and provides additional support for the trophic disruption hypothesis, emphasizing the need for proactive management and further research to mitigate the impacts of non‐native apex predators.

## AUTHOR CONTRIBUTIONS

**Olivia C. Hodgson:** Writing—original draft preparation; data curation; analysis; visualization; investigation. **Sydney Stark:** Investigation; writing—review and editing; data curation. **Megan K. Schall:** Resources; investigation; methodology; writing—review and editing; data curation; supervision; project administration; funding acquisition. **Geoffrey D. Smith:** Resources; investigation; methodology; writing—review and editing; data curation; supervision; project administration; funding acquisition. **Kelly L. Smalling:** Writing—review and editing; supervision; funding acquisition. **Tyler Wagner:** Resources, investigation, methodology, analysis, visualization, writing—review and editing, supervision, project administration, funding acquisition.

## CONFLICT OF INTEREST STATEMENT

The authors declare no conflicts of interest.

## Supporting information


Appendix S1.

## Data Availability

Data (Hodgson, Stark, Schall, Smith, & Wagner, 2024) are available via the United States Geological Survey (USGS) at https://doi.org/10.5066/P1DHTVLV. Code (Hodgson, Stark, Schall, Smith, Hopkins, et al., 2024) are available through the USGS at https://doi.org/10.5066/P16KW2QC.
