# Supplementary material for: Invasive predatory fish occupies highest trophic position leading to expansion of isotopic niches in a riverine food web
Source: Ecology. 2025 Sep 4;106(9):e70180. doi: 10.1002/ecy.70180 (PMC12411813; doi:10.1002/ecy.70180)
Supplement: Supplementary file 1 — Appendix S1. [file ECY-106-e70180-s001.pdf]

Appendix S1

Invasive predatory fish occupies highest trophic position leading to expansion of isotopic niches in a riverine food web

Olivia C. Hodgson, Sydney Stark, Megan K. Schall, Geoffrey D. Smith, Kelly L. Smalling, and Tyler Wagner

Journal: *Ecology*

Table S1: List of common and binomial names of prey fish species collected from sites invaded and not invaded by flathead catfish (*Pylodictis olivaris*) in the Susquehanna and West Branch Susquehanna Rivers, Pennsylvania. All species are native to the Susquehanna River, Pennsylvania study reaches, except for rock bass, which is naturalized (established for approximately 100 - 125 years). Data are available from Hodgson et al. (2024): https://doi.org/10.5066/P1DHTVLV.

**Flathead Presence**	**Species**
Invaded	Bluntnose minnow (*Pimephales notatus*)
	Central stoneroller (*Campostoma anomalum*)
	Rock bass (*Ambloplites rupestris*)
	Shorthead redhorse (*Moxostoma macrolepidotum*)
	Spotfin shiner (*Cyprinella spiloptera*)
	Spottail shiner (*Notropis hudsonius*)
	White sucker (*Catostomus commersonii*)
Not Invaded	Northern hog sucker (*Hypentelium nigricans*)
	Spotfin shiner (*Cyprinella spiloptera*)
	Spottail shiner (*Notropis hudsonius*)
	Unidentified *Cyprinella* spp. or *Notropis* spp.

Figure S1 (next page): Relationship between $\delta^{15}N$ and organism length (mm) across three fish species:(A) smallmouth bass (*Micropterus dolomieu*), (B) channel catfish (*Ictalurus punctatus*), and (C) flathead catfish (*Pylodictis olivaris*). Solid lines are posterior mean fitted lines and indicate $\delta^{15}N$ enrichment patterns in fish from non-invaded sites (without flathead catfish), while dashed lines represent enrichment patterns in fish from invaded sites. Shaded regions are 95% credible intervals and points depict individual sampled organisms.

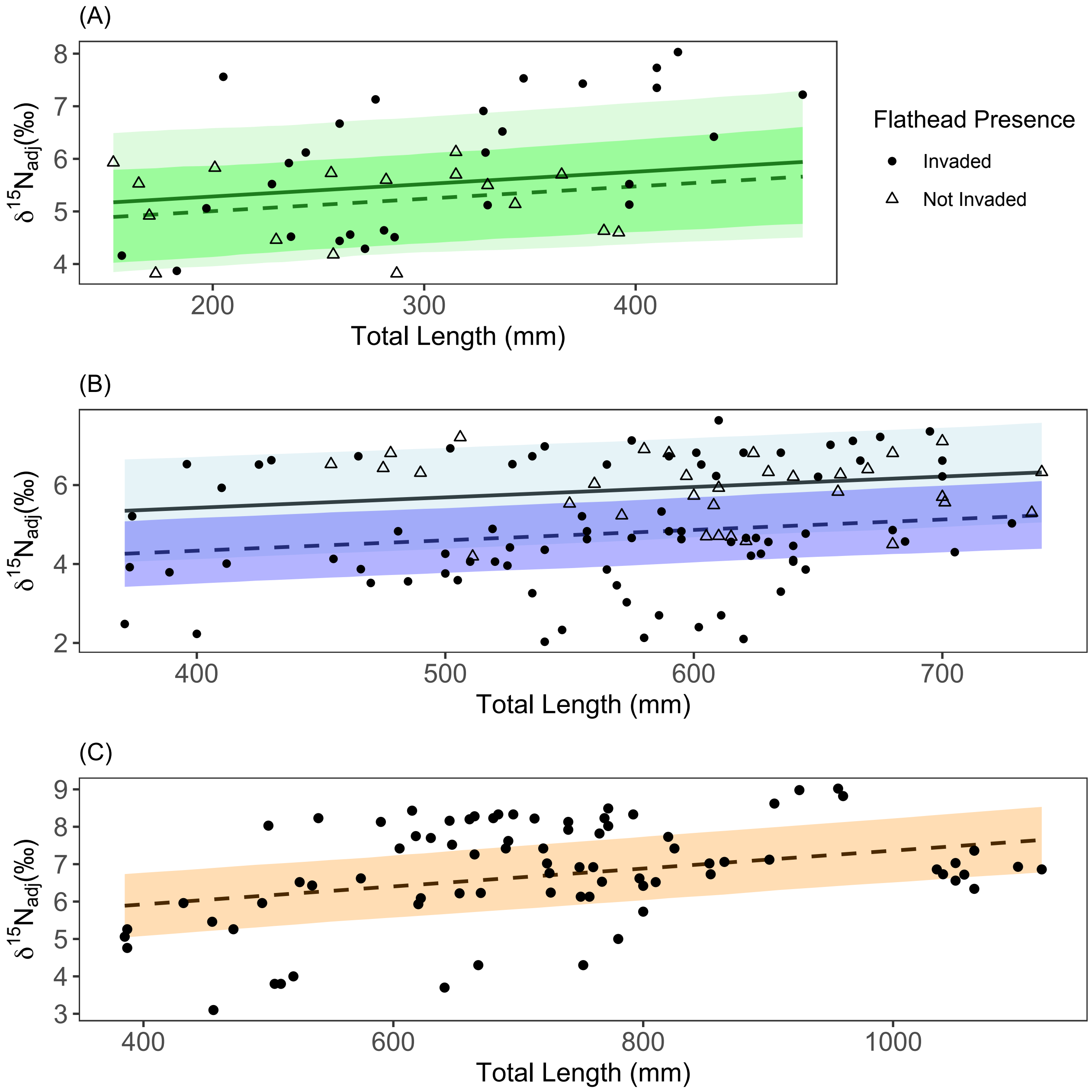
(A)
8
7
6
5
4
δ 15Nadj(‰)
200
300
400
Total Length (mm)
Flathead Presence
Invaded
Not Invaded
(B)
6
4
2
δ 15Nadj(‰)
400
500
600
700
Total Length (mm)
(C)
9
8
7
6
5
4
3
δ 15Nadj(‰)
400
600
800
1000
Total Length (mm)

Figure S1

References

Hodgson, O., S. Stark, M. Schall, G. Smith, and T. Wagner, 2024. Data describing a predatory fish invasion on a riverine food web. USGS data release. URL https://doi.org/10.5066/P1DHTVLV.
